# Emerging roles of type 1 innate lymphoid cells in tumour pathogenesis and cancer immunotherapy

**DOI:** 10.37349/etat.2024.00219

**Published:** 2024-04-23

**Authors:** James Michael Verner, Harry Frederick Arbuthnott, Raghavskandhan Ramachandran, Manini Bharadwaj, Natasha Chaudhury, Eric Jou

**Affiliations:** Sun Yat-Sen University Cancer Center, China; ^1^Robinson College, University of Cambridge, CB3 9AN Cambridge, United Kingdom; ^2^Medical Sciences Division, Oxford University Hospitals, OX3 9DU Oxford, United Kingdom; ^3^Balliol College, University of Oxford, OX1 3BJ Oxford, United Kingdom; ^4^Wexham Park Hospital, Frimley Health NHS Foundation Trust, SL2 4HL Slough, United Kingdom; ^5^Kellogg College, University of Oxford, OX2 6PN Oxford, United Kingdom

**Keywords:** Innate lymphoid cells, type 1 innate lymphoid cells, cytokines, innate immunity, tumour microenvironment, cancer therapy, preclinical models, immunotherapy

## Abstract

Innate lymphoid cells (ILCs) are the most recently discovered class of innate immune cells found to have prominent roles in various human immune-related pathologies such as infection and autoimmune diseases. However, their role in cancer was largely unclear until recently, where several emerging studies over the past few years unanimously demonstrate ILCs to be critical players in tumour immunity. Being the innate counterpart of T cells, ILCs are potent cytokine producers through which they orchestrate the overall immune response upstream of adaptive immunity thereby modulating T cell function. Out of the major ILC subsets, ILC1s have gained significant traction as potential immunotherapeutic candidates due to their central involvement with the anti-tumour type 1 immune response. ILC1s are potent producers of the well-established anti-tumour cytokine interferon γ (IFNγ), and exert direct cytotoxicity against cancer cells in response to the cytokine interleukin-15 (IL-15). However, in advanced diseases, ILC1s are found to demonstrate an exhausted phenotype in the tumour microenvironment (TME) with impaired effector functions, characterised by decreased responsiveness to cytokines and reduced IFNγ production. Tumour cells produce immunomodulatory cytokines such as transforming growth factor β (TGFβ) and IL-23, and through these suppress ILC1 anti-tumour actfivities and converts ILC1s to pro-tumoural ILC3s respectively, resulting in disease progression. This review provides a comprehensive overview of ILC1s in tumour immunity, and discusses the exciting prospects of harnessing ILC1s for cancer immunotherapy, either alone or in combination with cytokine-based treatment. The exciting prospects of targeting the upstream innate immune system through ILC1s may surmount the limitations associated with adaptive immune T cell-based strategies used in the clinic currently, and overcome cancer immunotherapeutic resistance.

## Introduction

Innate lymphoid cells (ILCs) are recently discovered innate immune counterparts of T cells with critical roles in various human immune-related pathologies including infection, autoimmunity and cancer [1–3]. ILCs are classified into five main subsets, namely group 1 ILCs which includes both natural killer (NK) cells and ILC1s, ILC2s, ILC3s and lymphoid tissue-inducer (LTi) cells, each with distinct roles in the protective immunity against a broad range of pathogens. Importantly, recent studies have begun to uncover that ILCs play important roles in modulating cancer development and progression, for example in the case of ILC2s, they may either promote lung and colorectal cancer (CRC) [4, 5], or contribute to anti-tumour immunity against pancreatic cancer [6]. On the other hand, the anti-tumoural effector functions of NK cells are well-established, and numerous modern immunotherapeutic strategies attempt to utilise NK cells to treat cancer in oncology clinical trials [7].

As alluded to, group 1 ILCs can be broadly divided into NK cells and ILC1s [8] based on a range of features such as their transcription factors, ontogeny, receptor expression and nature of their response [9–12]. Further characterisation is possible based on various organ-specific phenotypes and effector functions, with NK cells being cytotoxic while ILC1s are largely considered to be non-cytotoxic and instead exert effector functions through the production of cytokines such as interferon γ (IFNγ) [13]. Traditionally, NK cells are typically defined by the expression of eomesodermin (Eomes) and T-box expressed in T cells (T-bet) while ILC1s seldom express the former. Some studies have suggested that functionally, the maturation of NK cells requires the sequential activation of Eomes then T-bet [14]. In contrast, it has been proposed that ILC1s may express one of T-bet or in rarer cases Eomes, but not both [15]. Recently, a common group 1 ILC progenitor was identified, called aceNKP, which differentiates into a spectrum of group 1 ILCs, indicating that this group is formed of a continuum, rather than discrete populations of cells [16]. While unlike NK, B and T cells, ILCs are mainly tissue-resident cells [17], some migration does occur with ILC1s being the main migratory ILC subset [18].

Unlike NK cells, much less is known about ILC1s and their role in cancer. While direct cytotoxicity against tumour cells is one of the key defining features of NK cells, ILC1s are largely non-cytotoxic except under specific circumstances [2]. Nevertheless, ILC1s are potent producers of the type 1 cytokine IFNγ, which has well-established anti-tumoural functions [2], and patients with cancer that express a high IFNγ-related signature are associated with improved prognosis and response to immunotherapies including immune checkpoint inhibitors [19]. Accordingly, recent studies have found ILC1s to similarly exert protective immunity against certain cancer types including breast cancer and CRC as shown in animal models, and acute myeloid leukaemia (AML) in both experimental models and human patients [20–22]. ILC1s have been shown to infiltrate tumours upon cellular transformation in spontaneous murine cancer models, and exert cytotoxicity against tumour cells in response to interleukin-15 (IL-15) [23]. Importantly, the anti-tumoural functions of ILC1s and NK cells are non-redundant, as shown in a recent study on cancer metastasis to the liver, where the former was found to have a greater role in preventing metastasis seeding while the latter controls neoplastic growth [24]. Conversely, others have found that group 1 ILCs may contribute to the immunosuppressive tumour microenvironment (TME) under certain contexts limiting the anti-tumour immune response [25, 26]. Accordingly, one study proposed that the ratio of intra-tumoural ILC1s to NK cells may have an effect on tumour growth, with ILC1s having an inhibitory role against NK cell-mediated anti-tumour immunity [27].

Critically, most contemporary immunotherapies used in the clinic to date, in particular the revolutionary immune checkpoint inhibitors, largely focus on utilising T cells for cancer treatment, however only a minority (an estimated 12.46%) of patients respond [28, 29]. Better understanding of ILC1s, which lie upstream of T cells in the anti-tumour type 1 immune response, will be crucial in further enhancing T cell function and overcoming immunotherapeutic resistance [5, 30].

In this review, an overview of the current understanding of ILC1s in cancer is provided, together with the main ILC1-activating cytokines IL-12 and IL-15 in the context of ongoing clinical trials. The key factors that influence ILC1 function in cancer are summarised, alongside a discussion on potential strategies and prospects of harnessing ILC1s for future cancer immunotherapies.

## Overview of ILC1s in cancer

ILC1s being the innate counterparts to adaptive type 1 T helper (Th1) cells [31, 32], are activated by cytokines associated with stimulating the type 1 immune response such as IL-12 [33] and IL-15 [34, 35]. Following activation, ILC1s exert effector functions through the release of the cytokines IFNγ and tumour necrosis factor (TNF)α [11, 36, 37]. The overarching role of the type 1 immune response is the generation of protective immunity against intracellular pathogens and tumours [11]. Accordingly, both NK cells and ILC1s have been shown to exhibit anti-tumoural properties, however, more recent studies indicate that under certain contexts depending on the TME, ILC1s may elicit pro-tumoural effects and contribute to cancer progression. Factors that influence ILC1 function in cancer can be spatial or temporal, and are dependent on the cancer type involved, their location in the tumour tissue, the TME cytokine milieu and disease stage of the cancer. Furthermore, the functions of ILC1s differ at different stages of the tumoural response and thus may show completely opposite effects on cancer progression depending on the disease stage. Harnessing group 1 ILCs for cancer immunotherapy therefore is heavily dependent on the understanding of their intricate biology and the interaction with other cell types in the TME. A careful balance of utilising and enhancing their anti-tumour functions while suppressing their pro-tumoural effects through meticulous control of the cytokine milieu will be crucial for success.

## IL-12-ILC1-IFNγ axis

### ILC1-macrophage axis

Professional antigen presenting cells (pAPCs) such as macrophages are one of the major sources of IL-12 [38, 39], including in the TME. The effect of IL-12 on group 1 ILCs depends on the subtype and location. During *Toxoplasma gondii* intestinal infections, ILC1s exhibit a greater response to IL-12 stimulation than NK cells, due to a higher constitutive expression of the IL-12 receptor beta 1 (IL-12Rb1) subunit [11]. IL-12 acts through binding to the IL-12 receptor, resulting in the activation of the janus kinase (JAK) 2-signal transducer and activator of transcription (STAT) 4 signalling pathway [40, 41]. STAT4 promotes the expression of IL-12 target genes, in particular IFNγ resulting in the downstream elicitation of group 1 ILC effector function [42, 43].

**Figure 1:**
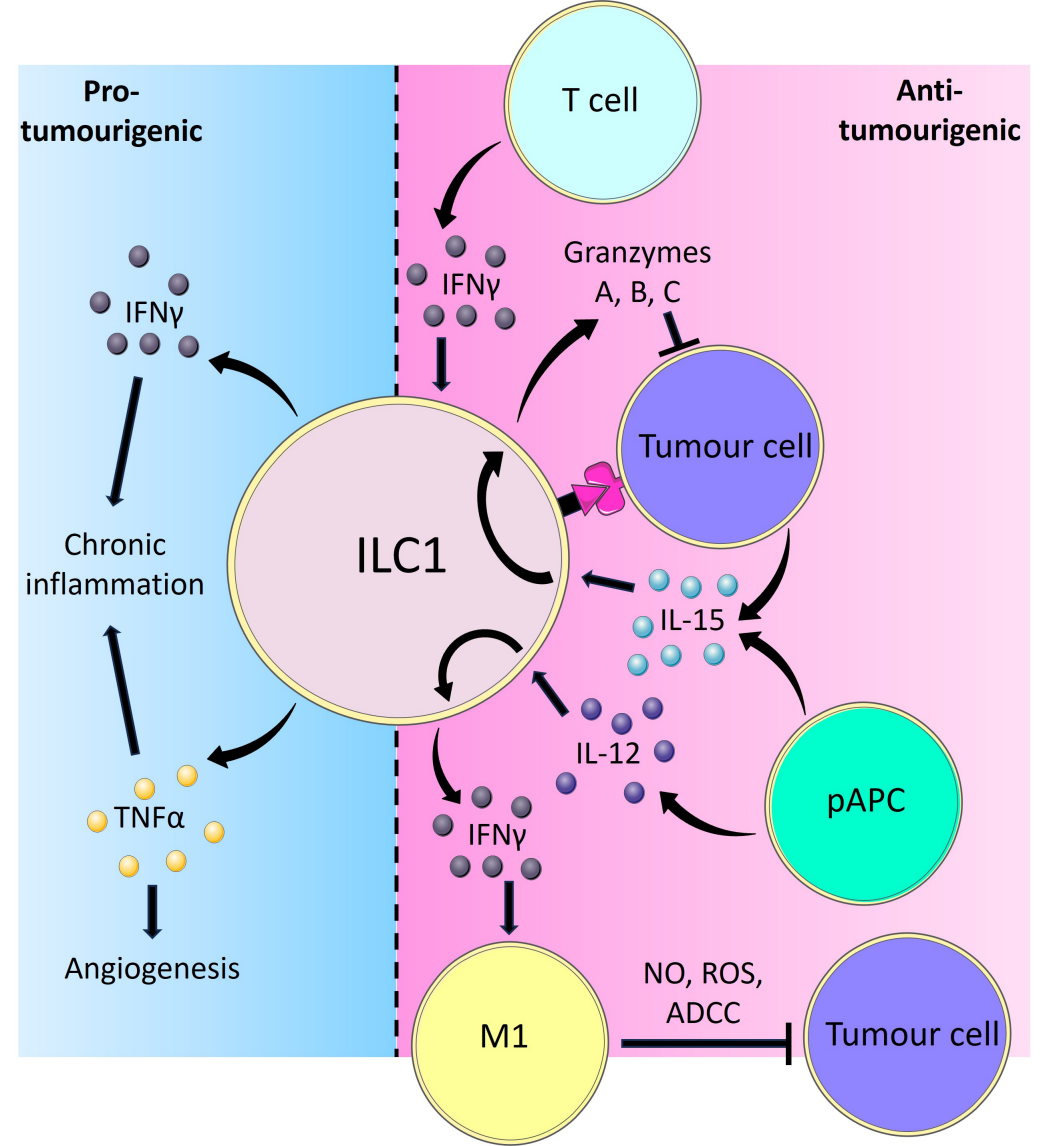
Overview of the pro-tumorigenic and anti-tumorigenic functions of ILC1s. ILC1s are activated by IL-12 and IL-15 derived from pAPCs and produce the cytokine IFNγ. IFNγ in turn causes TAMs to polarise towards a proinflammatory phenotype, which inhibits tumour cells through releasing NO and ROS, or via facilitating antibody-dependent cell cytotoxicity (ADCC). Tumour cells can also activate ILC1s through direct contact or IL-15. In response to IL-15, ILC1s adopt a cytotoxic phenotype and secrete GzmA, B, and C, which directly induce tumour cell death. Adaptive immune T cells are also potent producers of IFNγ, which reciprocally activates ILC1s to limit tumour progression. Part of the ILC1 response to tumours involves the release of pro-inflammatory cytokines IFNγ and TNFα. Whilst IFNγ and TNFα typically exert potent anti-tumoural effects, prolonged stimulation of ILC1s resulting in chronic inflammation by these cytokines may instead lead to cancer development. In such scenarios, TNFα is capable of inducing angiogenesis thereby facilitating tumour growth and metastasis formation [38, 44, 48, 61, 67, 78–82, 101, 102, 115–117]

It has been shown that ILC1-derived IFNγ reciprocally regulates macrophage activation [22]. Tumour-associated macrophages (TAMs) are found in solid tumours and classically exhibit one of two distinct phenotypes designated proinflammatory (formerly M1 macrophages) and alternatively activated (formerly M2) macrophages, reflecting their alignment towards type 1 and type 2 immunity respectively. The type 1 immune cytokine IFNγ promotes macrophage polarisation towards the anti-tumour proinflammatory IL-12-producing phenotype [44, 45], while stimulation by the type 2 cytokines IL-4 and IL-13 results in polarisation towards the tumour-promoting alternatively activated phenotype [46, 47]. This may result in a positive feedback loop where proinflammatory macrophage-derived IL-12 stimulates ILC1s to produce IFNγ, which in turn reinforces macrophage function (Figure 1). Mechanistically, proinflammatory macrophages have been shown to directly elicit cytotoxicity against tumour cells through releasing nitric oxide (NO) and reactive oxygen species (ROS), and via antibody-dependent cell-mediated cytotoxicity by recognising antibodies bound to tumour cells [48].

In the azoxymethane/dextran sodium sulphate (AOM/DSS) model of colitis-associated cancer (CAC), reverse transcription polymerase chain reaction (RT-PCR) showed there to be reduced IFNγ expression and flow cytometry showed there to be reduced ILC1 levels [22]. Additionally, ILC1 frequency positively correlated with proinflammatory macrophage populations in CRC tissue while negatively associating with alternatively activated macrophages [22]. This polarisation was blocked by the administration of anti-IFNγ antibodies, confirming that IFNγ is the key mediator of this process. These findings were corroborated by independent groups and indicate that ILC1s may control the ratio between proinflammatory and alternatively activated macrophages thereby promoting anti-tumour immunity. The protective effect of proinflammatory macrophages against CRC is further shown by others where stimulation of alternatively activated towards classical proinflammatory TAM polarisation through consuming a ketogenic diet can prevent the progression of CRC [49]. Furthermore, the significance of macrophage subtype ratio has also been demonstrated in lung cancer [50]. In peripheral blood samples from patients with lung adenocarcinoma compared to controls, there was an increase in alternatively activated macrophages while the proportion of proinflammatory macrophages decreased. Critically, the mRNA of both proinflammatory macrophages and importantly ILC1 cytokines were reduced while that of alternatively activated macrophages increased.

IFNγ-based treatment or adoptive lymphocyte transfer of ILC1s may thus provide another way of regulating the anti-tumoural macrophagic response (Figure 1). However, while promising, there are logistical challenges associated with ILC1 adoptive cell transfer, the most pertinent being to generate the sufficient numbers of ILC1s required to achieve clinical efficacy. ILC1s, like most ILC subsets, are relatively few in number compared to adaptive lymphocytes such as T cells, and therefore *in vitro* expansion of sort-purified ILC1s *ex vivo* or *de novo* generation of ILC1s *in vitro* may be required. In mice, recent studies have repeatedly demonstrated that adoptive transfer of flow cytometry sort-purified ILC1s can achieve efficacy experimentally, suggesting that ILC1s are able to retain their function *ex vivo* and after transfer, and that sufficient numbers to reach *in vivo* efficacy can be achieved [51, 52]. Indeed, recent studies have also demonstrated that bona fide human ILC1s can be generated and expanded *in vitro* from CD34^+^ haematopoietic stem cells (HSCs) through a combination of factors including IL-15 [53], which theoretically will provide sufficient numbers for human adoptive transfer therapies. The relative clinical efficacies of *in vitro*-generated and *ex vivo* ILC1s, along with the optimal cell numbers required will need to be established in future clinical trials.

A comprehensive list of ongoing clinical trials involving IL-12 and IFNγ has been summarised recently [54]. Due to the toxicity associated with high levels of systemic IL-12 [55], more advanced methods of cytokine delivery have been developed in an attempt to improve anti-tumour efficacy and reduce associated cytotoxicity. One study utilised genetically engineered myeloid cells to deliver IL-12 to metastatic sites, which reversed immune suppression and reduced both primary and metastatic tumour burden in mice [56]. Other recent developments include engineered cytokines in the form of membrane-anchored IL-12 expressed on T cells made possible through retroviral gene transfer, which allowed targeted delivery of IL-12 to tumours while reducing systemic exposure and toxicity [57]. Similarly, antibody-cytokine fusions containing an antibody component that targets specific tumour antigens have also demonstrated promising efficacy in preclinical studies [58]. Small molecule inhibitors have also been proposed to temporarily inhibit cytokine pathways to limit treatment-associated toxicities [59]. Future strategies targeting this axis may involve concomitant adoptive transfer of ILC1s together with IL-12 administration which may show potentiation in anti-tumour efficacy.

### Direct anti-tumour effect of IFNγ

ILC1s have also been shown to exert anti-tumoural properties against haematological cancers such as in AML [20]. Excessive differentiation of leukaemia stem cells (LSCs) into leukaemia progenitor cells results in the propagation of AML [60]. At low frequencies, ILC1s may prevent such differentiation and instead divert LSCs into non-leukemic cells. While high frequencies of ILC1s can directly induce apoptosis of LSCs [20] through ILC1-derived IFNγ in a JAK-STAT or phosphatidylinositol-3-kinase (PI3K)/AKT pathway-dependent manner. Additionally, murine AML ILC1s produced less IFNγ than those from healthy mice, suggesting that the antileukemic function of ILC1s is impaired in AML. Thus, targeting ILC1s as a cell-based source of IFNγ, in addition to their other anti-AML functions, may be a suitable immunotherapy approach for treating AML.

However, recent studies have found that in melanoma and lung cancer, ILC1s may show an exhausted phenotype characterised by programmed cell death protein 1 (PD-1) and TNF-related apoptosis-inducing ligand (TRAIL) expression, similar to exhausted T cells found in many cancer types [61]. Accordingly, despite an increase in the total ILC1 population representing a build-up of cells, there is an overall reduction in ILC1-derived IFNγ secretion, indicating a similar impairment in function akin to exhausted T cells [62]. While this allows solid tumours to escape ILC1-derived IFNγ-mediated killing, there are exciting prospects of utilising checkpoint inhibitors, in particular PD-1 blockers to restore ILC1 function and IFNγ production. Indeed, blocking other inhibitory receptors on ILC1s for example cytotoxic T-lymphocyte-associated protein 4 (CTLA-4), T cell Ig and ITIM domain (TIGIT) and T cell immunoglobulin and mucin domain-containing protein 3 (TIM3) may similarly enhance ILC1 anti-tumour activity [63, 64]. However, it is important to realise that ILC2s also express inhibitory checkpoint receptors such as PD-1, and therefore systematic administration of immune checkpoint inhibitors may simultaneously precipitate pro-tumoural functions of ILC2s particularly in cancer types such as CRC and lung cancer where ILC2s are found to promote tumorigenesis [4, 5, 65, 66]. Indeed, the lack of efficacy of immune checkpoint inhibitors in the majority of CRC patients as seen in clinical trials may potentially be due to concomitant stimulation of pro-tumoural ILC2s offsetting the beneficial activation of anti-tumoural type 1 lymphocytes.

Nevertheless, the revolutionary success of checkpoint inhibitors observed in the clinic to date across many cancer types may in part be due to revitalisation of ILC1s in addition to T cells, and highlights PD-1^+^ ILC1s as potential biomarkers in the future to stratify patient likelihood of response to immune checkpoint inhibitor treatment.

## IL-15-granzyme-ILC1 axis

The cytokine IL-15 is produced by a broad range of cells including pAPCs, stromal cells, endothelial cells and tumour cells, and acts through the IL-15 receptor (IL-15R) [67]. The trimeric IL-15R is composed of IL-15Rα, β and γ chains [68–70], and binding of IL-15 to its receptor causes the activation of JAK1 and JAK3 which phosphorylate STAT3 and STAT5 respectively [71–73]. These then go on to regulate gene expression predominantly associated with the anti-cancer response.

Like IL-12, IL-15 increases the expression of IFNγ [74] and acts synergistically with IL-12 to potentiate ILC1 effector function [75]. NK cell-development in the bone marrow requires the IL-15R [76, 77], while murine intraepithelial ILC1s have been found to develop independently of IL-15R [75]. In a murine model of breast cancer, hematopoietic and mesenchymal stromal cell sources of IL-15 are found to be dispensable for the maintenance and activity of ILC1s, while epithelial cancer cell-derived IL-15 is essential for ILC1s’ anti-tumour function [78]. While classically thought to be non-cytotoxic, IL-15 increases expression of granzyme (Gzm)A, B and C and may promote ILC1s towards a cytotoxic phenotype against tumours [78–82]. Mechanistically, GzmA causes lytic granule-mediated noncanonical apoptosis and pyroptosis [83, 84]. Intriguingly, comparison of chromophobe renal cell carcinoma (RCC; chRCC) to clear cell RCC (ccRCC) in human patients revealed that chRCC tumours express higher levels of IL-15, and this is reflected by the higher expression of GzmA in ILC1s compared to NK cells in chRCC, while the opposite is observed in ccRCC which express minimal levels of IL-15 [78]. Accordingly, human patients with chRCC were found to have significant tumour infiltration of cytotoxic GzmA-expressing cells that positively correlated with survival [78]. Single-cell RNA sequencing (scRNAseq) of human patient tumour samples revealed that these GzmA-expressing cells consist of two clusters of ILCs and were defined by high expression of the killer cell lectin-like receptor B1 (*KLRB1*) gene (which encodes for CD161), a marker known to characterise functional, proinflammatory NK cells that are responsive to innate immune cytokines [85]. Accordingly, one cytotoxic cluster was identified as NK cells, due to the expression of sphingosine-1-phosphate receptor 5 (*S1PR5*) and Kruppel-like factor 2 (*KLF2*), while the other cluster was identified as tissue resident ILC1s based on expression of integrin subunit alpha 1 (*ITGA1*) and zinc finger protein 683 (*ZNF683*). *Ex vivo* stimulation of ILC1s isolated from human patient RCC exerted direct cytotoxicity against tumour cells in a dose-dependent manner in response to IL-15 stimulation in experimental killing assays, and targeted deletion of cancer cell-derived IL-15 in experimental animal models *in vivo* impaired ILC1 cytotoxic effector function, evident through a reduction in GzmB and GzmC expression and enhanced tumour burden.

It has been shown that ILC1s lacking the IL-7 receptor exhibited inflammation-independent cytotoxic activity at steady state through GzmB expression, supported by constitutive mammalian target of rapamycin (mTOR) activation [12]. This role of mTOR contrasts the pro-tumoural role it is typically assigned in papers [86] and this complex signalling network should be considered when proposing immunotherapies. For example, rapamycin, an mTOR complex inhibitor, is regarded as an anti-cancer drug, yet it has been shown to downregulate GzmB expression in ILC1 [12]. Future studies could compare the effectiveness of rapamycin in reducing cancer progression in cancers that experience a strong GzmB ILC1 response and those that do not and comparing how the administration of rapamycin alters the lymphoid cell populations.

Similarly, disruption of GzmC expressing cells result in accelerated tumour growth [21]. In one study *in vitro* stimulation of ILC1s and liver NK cells with IL-15 showed that only ILC1s exhibited GzmC expression [21] and supporting this, cells that secreted GzmC did not express Eomes. Compared to healthy mammary glands, an increase in GzmC expression was noted in the PyMT model of breast cancer [21] and this suggests that part of the immune response to breast cancer may involve increased GzmC release by ILC1s eliciting cytotoxicity against tumour cells in a perforin dependent manner [87, 88].

Administration of IL-15 as a sole immunotherapy agent in a clinical trial resulted in a significant increase in the number of circulating group 1 ILCs [77], however, anti-tumour efficacy was limited due to the presence of inhibitory immune checkpoints which limit group 1 ILC function, a lack of tumour-specific targeting by the group 1 ILCs and lethal autoimmunity at high doses [21]. To utilise IL-15 in immunotherapy, soluble IL-15Rα/IL-15 dimers are undergoing clinical development to improve their pharmacokinetic properties [89]. Furthermore, there is the potential for combination treatment using exogenous IL-15 complexes with immune checkpoint inhibitors which may synergistically enhance group 1 ILC cytotoxicity and block inhibitory immune checkpoint pathways respectively, thereby potentiating treatment efficacy. These findings also have important implications in the development of ILC1-based adoptive cell transfer treatment strategies against cancer. A comprehensive list of all ongoing oncology clinical trials involving IL-15 is summarised for both solid (Table 1) and haematological (Table 2) cancers. Pre-activation of ILC1s with IL-12 and IL-15 prior to patient treatment may synergistically enhance ILC1 antitumour activity via increasing IFNγ and cytotoxicity, and could potentially circumvent the toxicity observed in clinical trials where potent cytokines such as IL-12 are directly administered to patients.

**Table 1:** A comprehensive list of ongoing oncology clinical trials involving IL-15 in solid tumours

**Clinical trial ID**	**Phase**	**Number of patients**	**Cancer type**	**Treatment**	**Status**	**Estimated study completion date**
NCT03388632	Phase 1	50	Solid tumour (metastatic or refractory)	Recombinant human IL-15 (rhIL-15) plus nivolumab or ipilimumab	Recruiting	December 2023
NCT05964361	Phase 1/2	10	Oesophageal, pancreatic, ovarian and liver cancer	IL-15-transpresenting Wilms’ Tumor-1 (WT1)-targeted dendritic cell vaccine	Not yet recruiting	September 2025
NCT05304936	Phase 1b/2	60	Advanced pancreatic carcinoma	HCW9218 [bifunctional transforming growth factor β (TGFβ) antagonist/IL-15 protein complex]	Recruiting	February 2025
NCT05620134	Phase 1/2	149	Various unresectable, locally advanced or metastatic tumour types	JK08 (IL-15 antibody fusion protein targeting CTLA-4)	Recruiting	February 2026
NCT05322408	Phase 1	24	Various advanced solid tumours	HCW9218 (bifunctional fusion protein complex of TGFβ receptor II (TGFβRII) domains, human tissue factor, and IL-15, with a second soluble fusion of TGFβRII domains and a domain of IL-15Rα)	Recruiting	January 2027
NCT05470283	Phase 1	30	Metastatic melanoma	OBX-115 [tumour infiltrating lymphocytes (TIL) engineered with membrane-bound IL-15 plus acetazolamide]	Recruiting	April 2027
NCT04294576	Phase 1	92	Locally advanced/metastatic tumours	BJ-001 (IL-15 fusion protein plus pembrolizumab)	Recruiting	October 2024
NCT06060613	Phase 1/2	32	Metastatic melanoma	OBX-115	Recruiting	October 2027
NCT03563157	Phase 1b/2	332	Metastatic CRC	NANT CRC vaccine plus a variety of interventions one of which is ALT-803 (recombinant human super agonist IL-15 complex)	Active	December 2022
NCT05419011	Phase 2	186	Lynch syndrome	Tri-Ad5 vaccines plus nogapendekin alfa (N-803)	Recruiting	February 2027
NCT04390399	Phase 2	328	Pancreatic cancer	Standard-of-care chemotherapy plus aldoxorubicin hydrochloride, N-803 and PD-L1 targeting-high affinity NK (t-haNK)	Recruiting	September 2024
NCT03387085	Phase 1/2	79	Triple-negative breast cancer	NANT triple-negative breast cancer vaccine plus various chemotherapy and immunotherapy agents (one of which is N-803)	Active	October 2023
NCT04290546	Phase 1	25	Squamous cell carcinoma of head and neck, and salivary gland carcinoma	CIML NK cell infusion plus N-803 plus ipilimumab or cetuximab	Recruiting	March 2024
NCT05327530	Phase 2	252	Locally advanced or metastatic urothelial carcinoma	Avelumab plus NKTR-255 (polyethylene glycol-conjugate of rhIL-15)	Recruiting	January 2025
NCT03022825	Phase 2/3	190	High-grade non-muscle invasive bladder cancer	Bacillus Calmette-Guerin (BCG) plus N-803 or N-803 only	Recruiting	October 2028
NCT05445882	Phase 2	28	Castration-resistant prostate cancer	N-803 or N-803 and BN-Brachyury or N-803 and bintrafusp alfa	Not yet recruiting	August 2026
NCT05676749	Phase 1	20	Metastatic NSCLC	C-TIL051 (TIL therapy) plus NKTR-255 plus pembrolizumab	Not yet recruiting	March 2027
NCT04659629	Phase 1	310	Refractory/relapsed solid tumours	NL-201 (IL-2/IL-15 agonist) or NL-201 plus pembrolizumab	Active	December 2024
NCT06083883	Phase 1	44	Synovial sarcoma and myxoid/round cell liposarcoma	NY-ESO-1 T cell receptor (TCR)/IL-15 NK (NK cells engineered to express TCR and IL-15) plus fludarabine plus cyclophosphamide	Not yet recruiting	November 2028
NCT05334329	Phase 1	21	NSCLC	Cord blood (CB)-NK (umbilical CB derived-NK cells) expressing soluble IL-15 (sIL-15) and PD-L1 plus atezolizumab plus fludarabine plus cyclophosphamide	Recruiting	September 2025
NCT06066424	Phase 1	54	Advanced solid tumours	Trophoblast cell surface antigen 2 (TROP2)-chimeric antigen receptor (CAR)-NK cells (IL-15-transduced) plus rimiducid plus fludarabine plus cyclophosphamide	Not yet recruiting	April 2040
NCT04377932	Phase 1	24	Paediatric solid tumours [glypican 3 (GPC3)-positive]	AGAR T cells (GPC3-specific CAR and expresses IL-15)	Recruiting	February 2040
NCT05103631	Phase 1	27	Solid tumours (GPC3-positive)	AGAR T cells	Recruiting	December 2039
NCT05703854	Phase 1/2	50	RCC, mesothelioma and osteosarcoma	CAR.70/IL-15-transduced CB-derived NK cells plus fludarabine plus cyclophosphamide	Recruiting	September 2027
NCT04715191	Phase 1	24	Paediatric solid tumours (GPC3-positive)	CARE T cells (GPC3-specific CAR and expresses IL-15 and IL-21)	Not yet recruiting	August 2041
NCT05922930	Phase 1/2	51	Ovarian cancer and mesonephric-like adenocarcinoma and Pancreatic cancer	TROP2-CAR-NK plus cyclophosphamide plus fludarabine	Not yet recruiting	August 2028
NCT05620342	Phase 1	24	Small and non-small cell lung carcinoma	Inducible caspase 9 (iC9).GD2.CAR.IL-15 T-cells (GD2-specific CAR and expresses IL-15 and iC9)	Recruiting	February 2027
NCT03721068	Phase 1	18	Neuroblastoma and osteosarcoma	iC9.GD2.CAR.IL-15 T-cells plus cyclophosphamide plus fludarabine	Recruiting	June 2039
NCT03294954	Phase 1	36	Neuroblastoma	GINAKIT cells (GD2-specific CAR and expresses IL-15)	Recruiting	August 2040
NCT05642195	Phase 1/2	30	Non-small-cell lung cancer	H1299 lung cancer vaccine with adjuvant alone or plus N-803	Recruiting	December 2035
NCT05396391	Phase 1a/1b/2a	140	Various unresectable solid tumours and refractory non-Hodgkin’s lymphoma*	IAP0971 (immunocytokine that binds specifically to PD-1 and fuses IL-15/IL-15Rα complex)	Recruiting	November 2024
NCT04261439	Phase 1/1b	60	Advanced solid tumours and lymphoma*	NIZ985 (recombinant heterodimer of IL-15 and IL-15Rα plus spartalizumab plus tislelizumab)	Active	November 2023

*: mixed studies where patients with both solid and haematological cancers were included

**Table 2:** A comprehensive list of ongoing oncology clinical trials involving IL-15 in haematological cancers

**Clinical trial ID**	**Phase**	**Number of patients**	**Cancer type**	**Treatment**	**Status**	**Estimated study completion date**
NCT05110742	Phase 1/2	48	Various haematological malignancies	CAR.5/IL-15-transduced CB-NK cells plus cyclophosphamide plus fludarabine	Not yet recruiting	December 2027
NCT04814004	Phase 1	20	B-cell malignancies	hCD19.IL-15.CAR-iNKT [invariant NK T (iNKT) cells expressing human CD19 (hCD19) CAR and IL-15]	Recruiting	April 2024
NCT03774654	Phase 1	48	Relapsed or refractory non-Hodgkin lymphoma, chronic lymphocytic leukemia (CLL), acute lymphocytic leukemia (ALL) and small lymphocytic lymphoma	CD19.CAR-allogeneic NK T (aNKT) cells (expresses CD19 antibody, CD28 and IL-15)	Recruiting	March 2035
NCT05487651	Phase 1	36	B-cell malignancies	CD19.CAR-aNKT cells (expresses CD19 CAR and IL-15)	Recruiting	December 2024
NCT05020678	Phase 1	150	B-cell malignancies	NKX019 (CAR NK expressing CD19 and membrane-bound IL-15)	Recruiting	December 2038
NCT04623944	Phase 1	90	AML and myelodysplastic syndromes (MDS)	NKX019	Recruiting	July 2039
NCT05359211	Phase 1	24	Large B-cell lymphoma (including diffuse large B-cell lymphoma, mediastinal large B-cell lymphoma and follicular lymphoma)	Anti-CD19 CAR T cells plus NKTR-255 plus fludarabine plus cyclophosphamide	Recruiting	December 2024
NCT05092451	Phase 1/2	94	B-cell lymphoma. MDS and AML	CAR.70/IL-15-transduced CB-NK cells plus fludarabine plus cyclophosphamide	Recruiting	August 2026
NCT02752243	Phase 1/2	32	MDS and acute leukaemia	IL-15-activated cytokine-induced killer (CIK) cells	Active	March 2024
NCT05618925	Phase 1	20	Non-Hodgkin lymphoma	CD19 t-haNK suspension plus N803 plus various chemotherapy agents	Not yet recruiting	September 2026
NCT06066359	Phase 1/2	44	Multiple myeloma	NY-ESO-1 TCR/IL-15 NK cells plus fludarabine plus cyclophosphamide	Not yet recruiting	August 2028
NCT05667155	Phase 1	48	B-cell non-Hodgkin lymphoma	CB dual CAR-NK19/70 (expresses anti-CD19/CD70 CAR and IL-15)	Recruiting	December 2025
NCT05182073	Phase 1	168	Multiple myeloma	FT576 [expresses B-cell maturation antigen (BCMA) CAR, high-affinity and non-cleavable CD16 (hnCD16) and IL-15/IL-15R fusion protein]	Recruiting	February 2040
NCT03774654	Phase 1	48	Relapsed or refractory non-Hodgkin lymphoma, CLL, ALL and small lymphocytic lymphoma	CD19.CAR-aNKT cells (expresses CD19 antibody, CD28 and IL-15)	Recruiting	March 2035

## Temporal factors on ILC1 function

Impaired ILC1 function is associated with cancer disease progression [90]. Using the AOM/DSS model of CAC, the temporal effect of cancer disease progression on ILC1s was examined by assessing tumour ILC1 phenotype and function at different stages of the disease [90]. In late-stage CAC, ILC1s were found to be reduced in frequency, and showed a decrease in IL-12 receptor expression and IFNγ production indicating impaired anti-tumoural effector function. Further phenotypic interrogation of these tumour ILC1s revealed an overall transition from expression of activating receptors [e.g., killer cell lectin-like receptor D1 (*Klrd1*), natural cytotoxicity triggering receptor 1 (*NKp46*), killer cell lectin-like receptor C2 (*Klrc2*), killer cell lectin-like receptor subfamily B member 1C (*Klrb1c*)] in early disease stage towards inhibitory receptors [e.g., killer cell lectin-like receptor family E member 1 (*Klre1*), killer cell lectin-like receptor subfamily A (*Klra*)] in late stage cancer. This transition towards an inhibited state is likely as a result of chronic stimulation, mirroring T cells whereby continuous tumour antigen stimulation leads to increased expression of the inhibitory receptors PD-1 and CTLA-4 and functional exhaustion [91]. Similarly, others have found that whilst in malignant melanoma there is a sizable intratumoural ILC1 population, overall production of IFNγ is reduced consistent with functional impairment [62].

These findings have important implications for immunotherapy, as the reduced responsiveness of ILC1s towards anti-tumoural cytokines such as IL-12 may limit the efficacy of IL-12-based immunotherapeutic strategies. Increasing the treatment dose of IL-12 administered to counter the reduction in IL-12 receptor expression is unlikely to be feasible given the toxicity observed with current dosages in existing clinical trials. Alternatively, while adoptive transfer of fresh activated ILC1s may overcome this, it is unclear with the current understanding whether transferred ILC1s will remain functional upon entering the TME and the numbers needed to transfer to achieve clinical efficacy. Further studies investigating the effect of chronic tumour stimulation on ILC1 functionality are required.

## Pro-tumoural roles of ILC1

### Chronic inflammation

Conversely, ILC1s may promote tumorigenesis depending on the disease stage and models used, typically under the circumstances of chronic inflammation. As alluded to previously, cancer progression is often associated with impaired ILC1 anti-tumoural function. Furthering this, independent groups have found that group 1 ILCs may indirectly promote cancer through inducing inflammation, one of the “hallmarks of cancer”, and predispose to cancer development [92, 93]. Crohn’s disease (CD) is one of the autoimmune inflammatory bowel diseases characterised by inflammation throughout the alimentary tract, and the associated chronic inflammation increases the risk of CRC in CD patients [94]. CD is known to be predominantly caused by a deranged type 1 immune response, and multiple studies have found ILC1s to play critical roles in the pathogenesis of CD driving inflammation and disease progression [25, 95–97]. Accordingly, ILC1 frequency is significantly elevated in the inflamed mucosa of CD patients [75]. Analysis of publicly available human CD scRNAseq databases allowed deciphering of the individual cell types’ contribution to the upregulation of CD-related genes [25]. Strikingly, ILC1s demonstrated the strongest induction of CD-related genes, and the particular genes involved were also highly enriched in both CAC and sporadic CRC suggesting that ILC1s play a critical role in intestinal inflammation and subsequent CRC development. Similarly, others have found inflammatory ILC1s to be increased at the precancerous stage of cutaneous squamous cell carcinoma (cSCC) contributing to tumour development and progression [26].

Mechanistically, ILC1s produce both IFNγ and TNFα. While IFNγ is largely anti-tumoural, TNFα can promote tumorigenesis for example through inducing angiogenesis [98–102]. Therefore, the ratios between the different cytokines ILC1s secrete and the overall tumour inflammatory milieu may contribute to determining their net effect on cancer development and progression. Within group 1 ILCs, NK cell populations exhibited a higher ratio of IFNγ^+^ cells to TNFα^+^ cells compared to ILC1s [36]. This provides an explanation for NK cells having greater anti-tumoural properties than ILC1s outside direct cytotoxicity. The involvement of ILC1s and TNFα in tumour development is further demonstrated through the observation that skin precancerous papilloma-associated ILC1 populations have the greatest ratio of TNFα^+^ cells to IFNγ^+^ cells, resulting in net pro-tumoural effects [26]. The reduction in IFNγ expression correlated with increased expression of inhibitory checkpoint receptors on ILC1s including PD-1 and TIM3 in both mice and human cSCC. Potential immunotherapeutic strategies to overcome this may involve combination treatment of immune checkpoint inhibitors to revitalise ILC1 function, together with cytokines such as IL-12 and IL-15 that stimulate ILC1 IFNγ production.

### Plasticity

**Figure 2:**
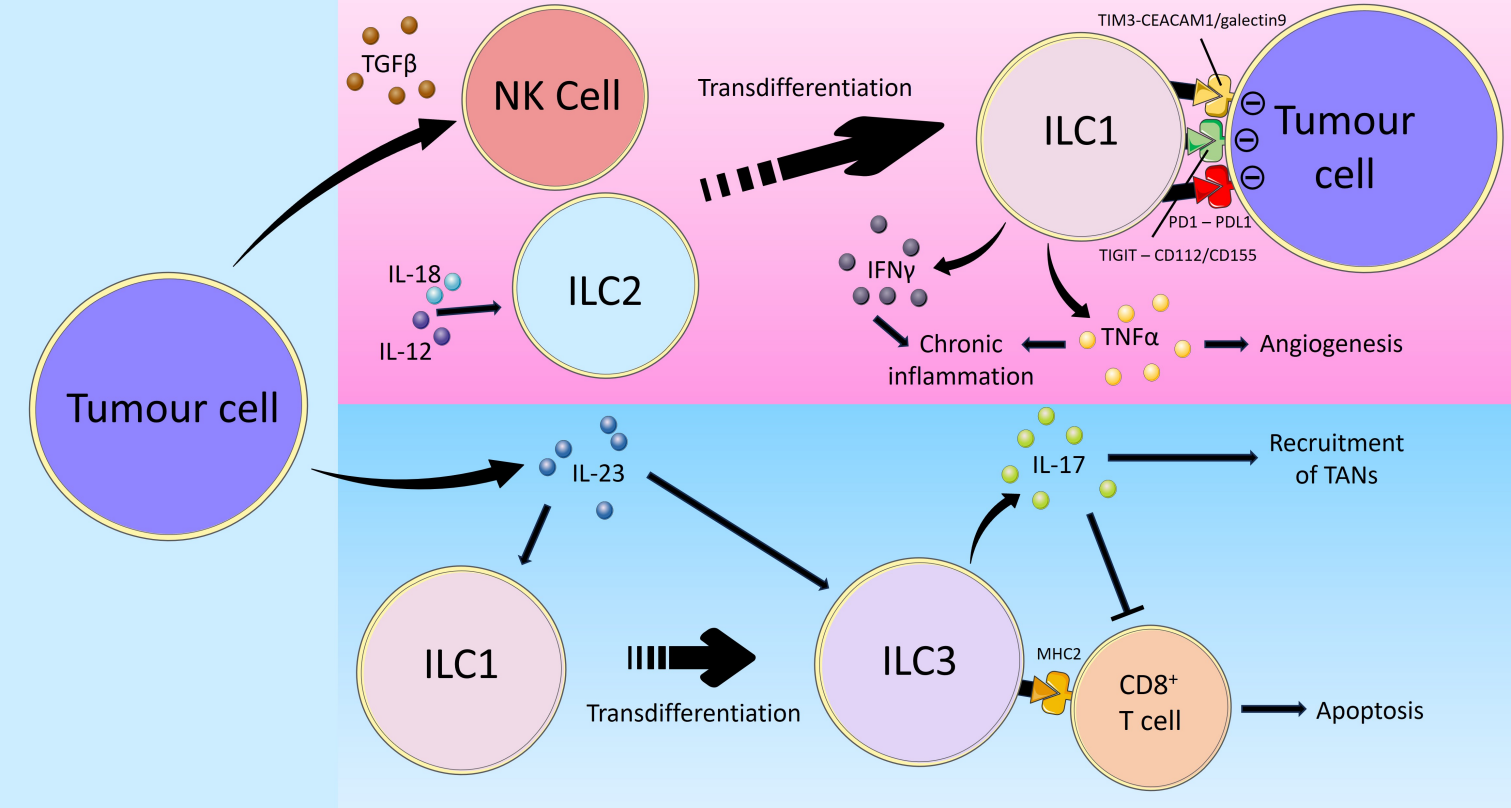
Overview of tumour immune-evasion via inducing ILC plasticity. Cancer utilises multiple mechanisms to evade group 1 ILC-mediated anti-tumour immunity. Firstly, tumour cells secrete TGFβ which promotes the transdifferentiation of NK cells into ILC1s, thereby limiting their cytotoxic activity. On the other hand, the cytokines IL-12 and IL-18 in the TME may similarly polarise ILC2s towards an ILC1-like phenotype. Tumour cells express the inhibitory checkpoint ligands CD112/CD155, PD-L1, and carcinoembryonic antigen-related cell adhesion molecule 1 (CEACAM1)/galectin 9, which interact with TIGIT, PD1 and TIM3 on ILC1s respectively, leading to impaired ILC1 effector function and reduced anti-tumoural activity. Furthermore, tumour cell-derived IL-23 promotes the transdifferentiation of ILC1s into ILC3s in addition to directly stimulating ILC3 activity. Unlike anti-tumoural ILC1s, ILC3s promote cancer progression through the secretion of IL-17. IL-17 recruits TANs and inhibits anti-tumoural CD8^+^ T cells. Certain ILC3s also express MHC-II which directly induce CD8^+^ T cell apoptosis, thus reducing the anti-tumoural immune response and facilitate cancer disease progression [36, 64, 100–102, 111, 112, 114, 115, 118–124]

ILCs show plasticity and are able to polarise towards different subsets depending on the overall cytokine milieu [1, 103, 104]. In viral infections and patients with chronic obstructive pulmonary disease (COPD), ILC2s have been documented to convert to an ILC1-like phenotype characterised by T-bet expression and IFNg production in response to the cytokines IL-1b, IL-12 and IL-18 [105, 106]. Critically, established tumours are able to co-opt ILC plasticity mechanisms to achieve immune evasion (Figure 2). IL-13^+^IFNγ^+^ ILC2s have been identified in patients with CD, suggesting that plasticity of ILC2s towards an IFNγ-producing ILC1-like phenotype may contribute to CAC development [107]. In a study on human hepatocellular carcinoma (HCC), a combination of bulk RNA sequencing, flow cytometry and scRNAseq of ILCs from non-tumour liver margin and tumour core was performed to investigate the role of cytokines in modifying ILC composition and the impact this had on prognosis [108]. The tests identified NK cells in the non-tumour tissue that could transition into tumour ILC1s and NK-like ILC3s when influenced by TGFβ. The non-cytotoxic ILC1s that the NK cells transformed into were unable to control tumour growth or metastasis. In addition to causing plasticity between group 1 ILCs, TGFβ may also cause group 1 ILCs to acquire angiogenic abilities, as has been observed in non-small cell lung cancer (NSCLC) where the CD56^+^CD16^–^ NK cell subset was associated with increased vascular endothelial growth factor (VEGF) expression [109]. In that study, supernatants from the NSCLC NK cells induced endothelial cell chemotaxis and the formation of capillary-like structures *in vitro*, facilitating cancer metastasis formation. Thus, by facilitating the secretion of TGFβ in its microenvironment, tumours utilise the plastic nature of ILCs to evade surveillance and destruction [36]. Taken together, the plasticity of group 1 ILCs may complicate the prospects of analysing them for diagnostic purposes or targeting them in immunotherapies. For instance, adoptive transfer of NK cells or activated anti-tumoural ILC1s may show limited effector function upon encountering TGFβ in the TME thereby limiting treatment efficacy. Concomitant blockade of TGFβ may be required and various immunotherapeutic strategies are actively being developed to target the TGFβ signalling pathway [110].

Alternatively, in cases where ILC1s exert anti-tumoural functions, cancers may inhibit ILC1 activity by promoting the conversion of ILC1s into ILC3s and this is associated with a poor prognosis [111]. Analyses of ILC subsets and cytokine expression revealed there to be low percentages of ILC1s and high frequencies of ILC3s in pulmonary squamous cell carcinomas (SqCC) but not in adenocarcinomas. This contrast is likely related to the lack of IL-23 expression by adenocarcinoma cells while, tumour cells from SqCCs produced IL-23 that promoted the conversion of ILC1s to ILC3s which produced IL-17, the latter capable of facilitating the recruitment of myeloid-derived suppressor cells (MDSC) and tumour-associated neutrophils (TANs) to the TME [112, 113] and inhibiting the CD8^+^ T cell response [114]. Similarly, in HCC, it has been shown that IL-23 expression promoted the differentiation of ILC1s into natural cytotoxicity receptor (NCR)-negative ILC3s characterised by a lack of NKp46 expression [114]. In addition to contributing to HCC progression through IL-17 expression, these ILC3s were found to directly regulate T-cell responses through expression of major histocompatibility complex (MHC) class II, which reduced CD8^+^ T-cell proliferation and directly induced apoptosis.

Thus, in order to achieve maximal efficacy with group 1 ILC-based immunotherapies, focussing on stimulating ILC1 effector function alone is unlikely to be sufficient. Strategies to maintain the anti-tumoural group 1 ILC phenotype and prevent plasticity in the TME, for example through concomitant blockade of TGFβ and IL-23, will be equally crucial.

## Conclusions

ILC1s are the newest identified member of the anti-tumour type 1 immune system with exciting prospects in cancer immunotherapy. Like T cells, ILC1s show an exhausted phenotype in tumours and as such, are likely in part responsible for the anti-tumour treatment efficacy of the widely used immune checkpoint inhibitors observed in clinic to date. Currently, several oncology trials are attempting to directly treat patients with the type 1 immune cytokines IL-12 and IL-15, however are largely limited by toxicities. Adoptive cell transfer of ILC1s has great promise in the treatment of both solid and haematological cancers, and can be further enhanced by pre-activation of ILC1s by IL-12 or IL-15, while mitigating the risk associated with direct cytokine administration. Treatment efficacy may be potentiated through combination treatment with immune checkpoint inhibitors or inhibitors against IL-23 and TGFβ, which will further boost ILC1 function and prevent plasticity into pro-tumoural phenotypes upon entering the TME. Further studies investigating the developmental origin behind the heterogeneity regarding the ILC1 response to cancer will be crucial in shaping future cell transfer treatment strategies to generate ILC1s with optimal anti-tumour activities for cancer treatment.

## Abbreviations

AML: acute myeloid leukaemia
CAC: colitis-associated cancer
CAR: chimeric antigen receptor
CD: Crohn’s disease
chRCC: chromophobe renal cell carcinoma
CRC: colorectal cancer
CTLA-4: cytotoxic T-lymphocyte-associated protein 4
Eomes: eomesodermin
GPC3: glypican 3
Gzm: granzyme
HCC: hepatocellular carcinoma
iC9: inducible caspase 9
IFNγ: interferon γ
IL-15: interleukin-15
IL-15R: interleukin-15 receptor
ILCs: innate lymphoid cells
JAK: janus kinase
LSCs: leukaemia stem cells
MDS: myelodysplastic syndromes
MHC: major histocompatibility complex
mTOR: mammalian target of rapamycin
NK: natural killer
NO: nitric oxide
NSCLC: non-small cell lung cancer
pAPCs: professional antigen presenting cells
PD-1: programmed cell death protein 1
RCC: renal cell carcinoma
ROS: reactive oxygen species
scRNAseq: single-cell RNA sequencing
STAT: signal transducer and activator of transcription
TAMs: tumour-associated macrophages
T-bet: T-box expressed in T cells
TCR: T cell receptor
TGF: transforming growth factor
TIGIT: T cell Ig and ITIM domain
TIM3: T cell immunoglobulin and mucin domain-containing protein 3
TME: tumour microenvironment
TNF: tumour necrosis factor

## Declarations

### Copyright

© The Author(s) 2024.
